# Well-Being and Mental Health of Siblings of Children with Intellectual Disability: a Longitudinal Twin-Family Study

**DOI:** 10.1007/s10882-025-10013-z

**Published:** 2025-03-21

**Authors:** Aline K. Honingh, Linda K. M. Veerman, Meike Bartels, Paula S. Sterkenburg

**Affiliations:** 1 Department of Clinical Child and Family Studies, Vrije Universiteit Amsterdam, Van Der Boechorststraat 7, Amsterdam, 1081 BT The Netherlands; 2 Department of Biological Psychology, Vrije Universiteit Amsterdam, Van Der Boechorststraat 1, Amsterdam, 1081 BT The Netherlands; 3 Bartiméus, Oude Arnhemse Bovenweg 3, Doorn, 3941 XM The Netherlands

**Keywords:** Well-being, Mental health, Intellectual disability, Siblings, Twins, Longitudinal study

## Abstract

Families of children with an intellectual disability (ID) face several challenges that also have consequences for the well-being and mental health of these children's siblings. Many factors contribute to the siblings' well-being and mental health, and several studies have called for replicating their research with longitudinal data. In the present longitudinal study, the well-being and mental health of siblings of children with ID (the ID-group) or typical development (the comparison group) were investigated, considering family factors as mediating and moderating factors. Data were obtained from the Young Netherlands Twin Register, focusing on twins aged 5 to 12 years. Multilevel modelling was used to investigate effects on three sibling outcome measures: well-being, externalizing behavior, and internalizing behavior. The externalizing behavior of the index child (the child with ID in the ID-group; randomly chosen in the comparison group) mediated the relation between the intellectual status (ID vs. no ID) of the index child and their sibling's internalizing behavior. Index child externalizing behavior, parental well-being, and parental monitoring had significant effects on sibling outcomes. Maternal monitoring moderated the relation between the index child's externalizing behavior and their sibling's externalizing behavior. Thus, this longitudinal study confirms and nuances some previously reported relations concerning sibling outcomes. The results imply that it is important that care organizations use a family systems approach, especially when a child has externalizing behavior problems. This study shows that there are different ways to mitigate the impact of a child's externalizing behavior on their sibling, such as through improved parental monitoring.

Various disabilities have psychological and behavioral impacts not only on the child with the disability, but also on their siblings and parents. During the past two decades, several studies have investigated the impact of having a sibling with a disability (Dyke et al., 2009; Giallo & Gavidia-Payne, 2006; Saxena & Adamsons, 2013), or specific disabilities, such as autism (Ferraioli & Harris, 2009; Tomeny et al., 2017) or intellectual disability (ID) (Hayden et al., 2019; Marquis et al., 2019). These impacts include positive as well as more difficult aspects (Blamires et al., 2024). Siblings for example express a warm and loving sibling relationship (Cebula et al., 2024) and show higher levels of prosocial behavior (Pinquart, 2023). However, families with a child with ID can face several challenges. For example, children with ID are twice as likely to have mental health problems, such as behavioral disorders, than other children, which also impacts family members (Totsika et al., 2022). In addition, caring for a child with ID is associated with parental stress, though variation exists in this group due to different factors, such as difficult child behaviors or ineffective parental coping strategies (Biswas et al., 2015). Furthermore, disability in families correlates with socioeconomic stress (Farrell & Krahn, 2014) which can increase the stress that is experienced. Finally, siblings of children with ID can experience negative emotions (Yaldız et al., 2021), have behavioral and emotional problems (Hayden et al., 2019) and affected quality of life (Moyson & Roeyers, 2012).

In research focusing on siblings of children with ID, well-being and mental health are concepts that are often used to describe siblings’ functioning (Wakimizu et al., 2020; Wolff et al., 2022). Well-being can be assessed with measures representing concepts such as quality of life, happiness, satisfaction with life, and life fulfillment, which represent the affective and cognitive evaluation of one's life (Bartels & Boomsma, 2009). Sibling mental health has been referred to in terms of psychiatric disorders or clinical diagnoses (Wolff et al., 2022) but has also been described as emotional and behavioral functioning (Marquis et al., 2019). Emotional and behavioral functioning can, for example, be measured with the Strength and Difficulties Questionnaire (Goodman, 1997) and the Child Behavior Checklist (Achenbach, 1991), and is also referred to as internalizing behavior (e.g. sadness) and externalizing behavior (e.g. aggression).

The impact of having a sibling with ID on well-being and mental health has been partly explained by, or is related to a number of factors, many of which appear to be inter-related (Marquis et al., 2019; Wolff et al., 2022). Contributing factors can be organized into different frameworks, such as a bioecological framework (Saxena & Adamsons, 2013) and the Siblings Embedded Systems Framework (Kovshoff et al., 2017), including for example microsystems (e.g. family, school, neighborhood), macrosystems (e.g. laws, social policy) and chronosystems (e.g. change in beliefs, life stage). In this study we focus on the influence of family factors and characteristics of the child with ID, taking into account changing factors through time.

First, regarding the characteristics of the child with ID, behavioral problems were found to have a negative impact on the well-being and mental health of the sibling (Arnold & Heller, 2018; Marquis et al., 2019) and may even mediate the relation between ID and sibling impact (Neece et al., 2010). However, whether this mediation still holds in a longitudinal setting that accounts for variation between children is unclear.

Second, regarding family factors, parental well-being is of importance to sibling outcomes (Marquis et al., 2019). Parental well-being relates to the well-being of children (Newland, 2020) and has been expressed as a measure of parental stress (Neece et al., 2012), caregiver's burden (Wakimizu et al., 2020), or parental health (Marquis et al., 2019). Parental well-being has a bi-directional relation with child behavior problems (Neece et al., 2012). Moreover, Hayden et al. (2019) concluded that group differences between siblings of children with and without ID are no longer significant when family factors, such as primary carer psychological distress, are considered. However, it is not clear if and to what extent parental stress or well-being could explain the relation between a child's ID and their sibling's well-being or mental health.

Third, parenting behavior or style is another family factor that relates to the family system and plays an important role in sibling outcomes in families with a child with a disability (Giallo & Gavidia-Payne, 2006). For example, authoritative (as opposed to authoritarian) parenting was found to be positively related to cooperative behavior of the siblings (Platt et al., 2014). In families without disability, positive aspects of parenting have even been found to act as a protective factor or buffer, such as against the impact of low peer acceptance (Zarra-Nezhad et al., 2019) or family poverty (Brown et al., 2020). One aspect of the multifaceted construct of parenting (Sleddens et al., 2014) that is positively associated with child outcomes is parental monitoring (Chassin et al., 1993; Dick et al., 2007), which measures the degree to which the child's activities are supervised. It has not yet been investigated how parental monitoring can influence sibling well-being and mental health in a family with a child with ID and whether this can act as a buffer against behavioral problems of the child with ID, or parental stress.

Finally, additional factors that relate to sibling well-being and mental health include for example the age and gender of the siblings, birth order, the relationship between the siblings (Wakimizu et al., 2020), socio-economic status (SES) of the family, and whether or not there is a single-parent household (Hayden et al., 2019). Therefore, it is important to control for these factors as much as possible in a study of sibling well-being and mental health.

Twin pairs form a special group of siblings in which one of the above factors, namely birth order, is automatically controlled for because twins are of the same age. Therefore, focusing on twin pairs allows for a sibling study in which there is no age asymmetry in the sibling relationship. In twin studies, the term co-twin is used to indicate the birth partner of a (specific) twin. Twin pairs have rarely been studied in research on typically developing siblings/co-twins of children with special needs. We know of only one such study, which reported that these co-twins scored higher on cognitive empathy (Rum et al., 2022). As the relationship between siblings is positively related to the sibling's quality of life (Wakimizu et al., 2020) and the relationship between twins was found to be closer than between non-twin siblings (Fortuna et al., 2010), a sibling study with twin participants may show higher overall sibling well-being than a sibling study with non-twin participants. Within a sample of twin pairs, monozygotic (genetically identical) twins have been found to have a closer sibling relation than dizygotic twins (Fortuna et al., 2010). Therefore, in a study on sibling well-being among twin pairs, zygosity needs to be controlled statistically or methodologically.

Most sibling studies focus on a single point in time, revealing little about how sibling well-being and mental health changes over time (Farrell & Krahn, 2014; Saxena & Adamsons, 2013). In addition, several cross-sectional studies have expressed the need to replicate their results in a longitudinal study to validate the results over time (Hayden et al., 2019; Williams et al., 2022).

The aim of the present study is to build on the literature of well-being and mental health of siblings of a child with ID in a family perspective with twin pairs, using a longitudinal approach. We measured the well-being, internalizing, and externalizing behaviors of co-twins of children with ID over time between the ages of 5 and 12 years. Hereby we investigated the interaction with externalizing behavior of the child with ID, parental well-being and parental monitoring. Our first research question is: How does the well-being, internalizing, and externalizing behavior of co-twins of children with ID change over time from age 5 to 12 years of age? We expect well-being to decrease over time and internalizing and externalizing behavior to increase. Our second research question is: Does the intellectual status of a child (ID vs no ID) predict the well-being and behavioral problems of their sibling and, if yes, is this relation mediated by either the externalizing behavior of the child with ID or the well-being of the parents? We expect both behavioral problems and parental well-being to act as mediators. The third research question is: Given any effects of externalizing behavior of the child (with ID) and parental well-being on the sibling outcomes, can parental monitoring act as a buffer, in other words, is parental monitoring a moderator? We expect that more parental monitoring decreases the impact of child behavior problems and parental stress on sibling well-being and mental health.

## Method

This study is a longitudinal analysis of existing data of twin pairs aged 5, 7, 10, and 12 years, from the Young Netherlands Twin Register (YNTR; van Beijsterveldt et al. (2013)). The Netherlands Twin Register (NTR) has been collecting data on Dutch twin pairs since 1987 using paper, and later digital, questionnaires. Young twin pairs that participate in the YNTR are registered by their parents, who are recruited with the help of professional baby organizations that visit parents of newborns (van Beijsterveldt et al., 2013). Participating parents of twins receive questionnaires on a variety of topics when their twins are 1, 2, 3, 5, 7, 10, and 12 years old. Further details on the data collection of the full YNTR and participation rates can be found in van Beijsterveldt et al. (2013) and Bartels et al. (2007b).

### Data Selection Procedure

**Fig. 1:**
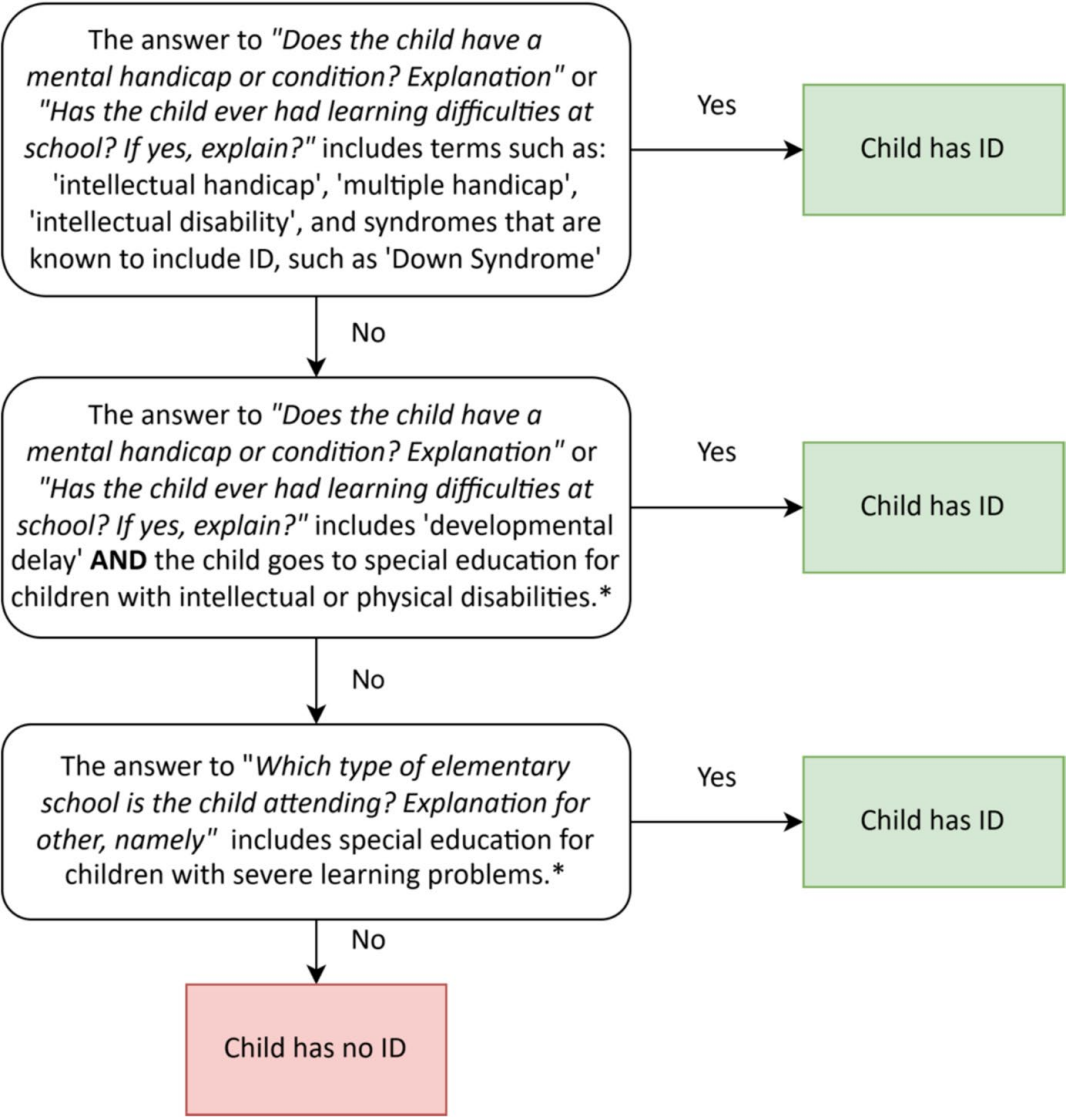
Flow Diagram of Selection Procedure of Children with an Intellectual Disability (ID)**.** *The YNTR includes several questions about learning problems and the type of education that is attended, relating to different school settings for children with ID, which have changed over time. The ‘severe learning problems’ from condition 3 relates to previous Dutch special education system (“ZMLK”) for children with ID, that later became part of the present special education system (“cluster 3”) for children with intellectual or physical disabilities (mentioned in condition 2)

For the present study, we selected a sample of twin pairs from the YNTR in which one twin, referred to as the index child, has an intellectual disability (ID) and the co-twin has no ID or other severe disabilities or disorders. The classification of ID was based on the mother's reports indicating that the child has a ‘mental handicap’, together with extra information on the type of handicap, other conditions, and education. The detailed selection process of children with ID can be found in the preregistration of the study (Honingh et al., 2022). The main steps are visualized in Fig. 1.

The co-twins of the children with ID were screened using the same variables to determine whether the co-twin had ID or another severe disorder or disability. Twin pairs were excluded when it was clear that or uncertain whether the co-twin had ID, or when the information indicated that the co-twin had a severe disability. However, to remain close to the population characteristics of siblings of children with neurodevelopmental conditions in which divergent neurocognitive profiles were found (Wolff et al., 2023), co-twins with *mild* neurodivergence were contained in our sample. For example, siblings with dyslexia or ADHD (symptoms), that parents did not indicate as being a disability, were contained in the sample. We included siblings with mild physical conditions (e.g., clubfoot, asthma or allergies) as well. We excluded those with more severe conditions, and those who attended special education.

The selection procedure resulted in 209 pairs of twins (referred to as the ID-group) from whom information was obtained on (at least one of the) ages 5, 7, 10, or 12 years. Not all variables are measured at all ages. Besides the 'well-being' variables, which are measured at ages 5 to 12, all used variables are only measured at ages 7 to 12. Therefore, two separate datasets were created: one containing data in the age range 5–12 (which is only used for the research question concerning the change in well-being over time), and one containing data in the age range 7–12 (which is used for all other research questions). A comparison group of children with typical development (TD), twin pairs in which both children have a typical development (referred to as the TD-group or comparison group) was selected for the age range 7–12. These children were automatically selected from the YNTR-data by choosing the answer 'no' to all the questions concerning a 'mental handicap', 'physical handicap', 'chronic condition or disability' and 'condition or handicap that seriously interferes with activities of daily living'. An overview of the samples we used can be found in Table 1.

**Table 1:** Available data in used samples per age group: intellectual disability vs. typical development

	Number of twin pairs	age 5	age 7	age 10	age 12
ID-group 5–12 (well-being)	87	21.8%	24.1%	41.4%	58.6%
ID-group 7–12	176		70.5%	60.2%	57.4%
TD-group 7–12	14,815		72.7%	66.5%	54.1%

It can be observed that for the 7–12 group, the sample sizes decrease for older children. This is due to two main reasons. First, not all twin pairs participating in the YNTR have reached the age to receive a specific questionnaire. For example, our data selection may contain twin pairs who are currently 6 years old and only completed the questionnaire at 5 years old. Second, some families did not participate in each wave of the study (drop-out), because of, for example, moving to a new address without notification (Bartels et al., 2007b). For the 5–12 group, the sample sizes increase with age. This is because this particular dataset is limited by the prevalence of the variable 'well-being'. The question about well-being was introduced in the YNTR at a later stage, when several twin pairs participating in the YNTR-study had reached a higher age (then 5). For example, twin pairs that received a first question on well-being at age 12, have entered a response at that age, but information on their well-being at previous ages was not collected.

One could wonder whether more drop-out is present in the ID-group than in the TD-group. To answer this question, bootstrapping was used to calculate a 95% confidence interval around the data percentages of the TD-group (Table 1), using the sample size of the ID-group (*n* = 176). Resampling 10000 times resulted in the following stable confidence intervals for age 7 [65.9, 79.0], age 10 [59.7, 73.3], and age 12 [46.6, 61.4]. The percentages of available data in the ID-group all fit within these 95% confidence intervals, meaning that there is no significant difference in the drop-out rate between the two groups.

### Participants

Table 2 shows the demographic characteristics of the ID-group (age 7–12) and the TD-group (age 7–12). In the ID-group, the child with ID is referred to as the index child. In the TD-group, one of the children was randomly selected as the index child. In both groups, the other child is referred to as co-twin.

**Table 2:** Demographics of the ID-group (7–12) and TD-group (7–12)

	Intellectual disability (*N* = 176)		Typical development (*N* = 14,815)		*X*^*2*^/*t*^*b*^
Characteristic	Mean/ Percentage	*(SD)*	Mean/ Percentage	*(SD)*	
*Children*					
Gender (% boys)	55.1		48.7		**2.60**
*Specification of ID*					
% Downsyndrome	6.3				
% Additional ASD	30.1				
% Additional ADHD	25.0				
% Additional physical					
/sensory disability	31.0				
*Co-twins*					
Gender (% boys)	51.7		48.6		**0.54**
*Parent/family*					
Marital status at age 12 (%					
divorced)	15.3		9.2		7.02^**^
SES^a^ at age 10	3.1	1.1	3.4	1.0	3.34^**^
*Other*					
Zygosity of twins (%					
monozygotic)	10.2		37.6		54.66^***^
Year of birth	1997	5.6	1998	7.0	2.96^**^

For a complete description of the dataset, some specifications and additional disorders (Autism Spectrum Disorder—ASD, Attention Deficit Hyperactivity Disorder—ADHD) are given for children in the ID-group

^a^Family socioeconomic status (SES) was based on the highest parental score within a family and was measured on a scale from 1 (profession on unskilled level) to 5 (profession on level scientific education)

^b^Chi squares in boldface

^**^p < .01. ^***^p < .001

### Measures

#### Child Behavior Checklist (CBCL)

The externalizing behavior of the child with ID and the externalizing and internalizing behavior of the co-twin without ID were assessed with the CBCL (Achenbach, 1991) at ages 7, 10, and 12 years, using maternal ratings. The Externalizing Behavior Scale is a broadband scale consisting of 35 items that includes the subscales Rule Breaking Behavior and Aggressive Behavior. The Internalizing Behavior Scale, consisting of 32 items, includes the subscales Withdrawn/Depressed, Anxious/Depressed, and Somatic Complaints. The questions in the CBCL are scored using a 3-point Likert-scale: 0 (not true), 1 (somewhat or sometimes true) and 2 (very true or often true). The scores on the Externalizing Behavior Scale and Internalizing Behavior Scale are the sums of the scores to the individual questions in the subscales. The CBCL is a widely used parent-report scale for measuring socio-emotional and behavioral functioning and has high reliability and validity (Albores-Gallo et al., 2007).

#### Well-Being

In the dataset that we used from the YNTR, well-being is measured with the Self-Anchoring Ladder (Cantril, 1965), which has a 2-year test–retest reliability of *r* = 0.65 (Palmore & Kivett, 1977). Well-being is measured with the question: 'Where on the bar would you say your life generally stands? The 10 means the best life you can imagine.' The outcome of this question is a number between 0 and 10. Well-being is separately measured for mothers, fathers, and each member of the twin-pairs. For the well-being score for children, the mother's ratings have been used.

#### Parental Monitoring

Parental monitoring is a dimension of parenting (Sleddens et al., 2014) that reports on the degree to which parents know the activities and view of the child (Chassin et al., 1993; Dick et al., 2007). In the YNTR, parental monitoring is measured for mothers and fathers at ages 7, 10, and 12 years by four statements in response to the question, 'How do you describe your way of parenting?': (1) “I always know where and with whom my child is if he/she is not at home,” (2) “I know the interest of the child and what kind of hobbies he/she likes,” (3) “I know the daily activities of the child,” and (4) “I take into account the view of the child.” Answers are given on a 4-point scale, from "seldom or never" to "almost always". The score for this variable is obtained by summing the individual scores from the separate statements. In the YNTR, the variable is obtained for both twins. This study focuses on outcomes for the co-twin without ID. Therefore, we have included the parental monitoring variable with respect to this co-twin. The father and mother ratings result in separate variables. The internal consistency measured by Cronbach’s alpha ranged from 0.42 to 0.80 depending on age and mother/father ratings, resulting in a mean of 0.61, which approaches the interpretation of “fair” for a 4-point scale (Ponterotto & Ruckdeschel, 2007).

### Analyses

To analyze our longitudinal data, we used multilevel modelling in R version 2022.02.3 with the nlme package (Pinheiro et al., 2023) using maximum likelihood estimation. Multilevel modelling enables estimation of change over time while taking into account the hierarchical nature of the data: multiple time points (level 1) nested in people (level 2). Multilevel models can handle missing items, due to the format of the data (long format) that is required. Therefore, it is not a problem if the number of available measurements is not the same for all individuals (Hox, 2010). Missing values in level 2 predictors might cause large deletions (Van Buuren, 2018), but since we have manually selected participants based on the intellectual status of the index child/ co-twin, this specific type of missingness does not apply to our data.

A number of variables in the dataset are skewed, causing a violation of the assumption of linearity. A commonly used approach in case of skewness is transformation of the data. However, transformations fundamentally change the nature of variables, making interpretation more complex (Osborne, 2002). When visually inspecting the separate relations contributing to the proposed models, it became clear that a linear model is appropriate for approximately 90% of the data and that the tails of some distributions cause deviation. Therefore, we chose not to transform the data, as a log-transformation would enlarge these deviations, but to stay aware that the results of the analyses do not apply to values in the tails of the distributions (e.g., for very high values of externalizing behavior).

#### Research Question 1: Change Over Time

The change over time of co-twin well-being (at ages 5, 7, 10 and 12), and co-twin externalizing behavior and internalizing behavior (at age 7, 10 and 12) in the ID-group were separately modelled using multilevel analysis. Multilevel modelling enables calculating *between variation*, the amount that people vary compared to one other, and *within variation,* the amount that people vary relative to their own average or trend. Using these concepts, the intraclass correlation (ICC) is calculated as: ICC = between variation/(between variation + within variation), which expresses the proportion of the total variance that is accounted for by the clustering of times points in people.

For each outcome variable (co-twin well-being, internalizing behavior, externalizing behavior), we compared different models. An overall change function was fitted to the sample, where the intercept and the slope were allowed to vary across individuals (defined as random coefficients). We started with the most simple model (m0) with a fixed and random intercept to calculate the ICC, which gives an indication of how much variation there is between people. We then added time (age) as predictor, first as fixed effect only (m1), which allowed us to calculate the average rate of change of the outcome variable. When time is added as random effect, the model (m2) takes into account varying values of the outcome variable over time which can be different for each person. An autoregressive covariance structure (autocorrelation structure of order 1 with a continuous time covariate, *r* = 0.2) was added in the last step (m3). The models (m0, m1, m2, m3) were compared using ANOVA.

#### Research Question 2: Group Difference and Mediation

To estimate whether the intellectual status of a child (ID vs TD) predicts the well-being and behavioral problems of their co-twin (first part of research question 2), the datasets of the two groups (the ID-group and the TD-group) were merged and a variable was added to indicate the intellectual status of the index child. As multilevel analysis was used, all predictors (including categorical predictors and covariates) were centered using grand mean centering (Yaremych et al., 2021). For each separate outcome variable (co-twin well-being, internalizing behavior, externalizing behavior), the model was built up in the same way as before (m0 to m3), and elements were removed again if not contributing significantly to the model. The covariates were then added to the model as fixed effects, after which the intellectual status of the index child (ID vs TD) was added to the model as a predictor. If this was a significant predictor for one of the outcome variables of the co-twin (well-being, internalizing behavior, externalizing behavior), the next question was whether this relation was mediated by the externalizing behavior of the index child, maternal well-being or paternal well-being (second part of research question 2). Indirect effects were estimated with multilevel mediation (Hayes & Rockwood, 2020), by calculating the separate paths in the mediation model. The following separate paths were calculated: the relation between predictor and mediators (path a), relation between mediators and outcome (path b), relation between predictor and outcome (path c), and the relation between predictor and outcome via the mediators (path c’). Hence, the indirect effect was calculated as *ab* and equals *c–c’*. Confidence intervals were calculated using a Monte Carlo method (using 20,000 repetitions) for multilevel mediation (Bauer et al., 2006; Selig & Preacher, 2008).

#### Research Question 3: Moderation

A multilevel moderation analysis was used to investigate the relations between the predictors parental well-being and externalizing behavior of the index child, and co-twin outcome variables well-being and mental health, with parental monitoring as moderator. We started with the same regression as before, where we modelled the sibling well-being/mental health against time with an autoregressive covariance structure. Elements that did not contribute significantly to the model were removed again. After adding the covariates, the predictors were added and, thereafter, the (possible) moderator. In the final step, the interaction with maternal/paternal monitoring was added to test for moderation. To test for robustness of the results concerning the random component (remember that in the comparison group, one member of a twin pair was randomly selected to be the index child), we repeated the analysis a hundred times.

## Results

The observed mean scores for the outcome variables, mediators, and moderators are provided in Table 3 for the ID and TD group for all ages.

**Table 3:** Descriptives of outcomes, mediators and moderators

	Intellectual disability			Typical development		
	*N*	range	mean (*SD*)	*N*	range	mean (*SD*)
Co-twin well-being						
Age 5	19	7–10	8.74 (0.87)	N/A*	N/A*	N/A*
Age 7	21	7–10	8.52 (0.87)	2862	4–10	8.51 (0.90)
Age 10	36	4–10	8.39 (1.20)	4371	2–10	8.46 (0.94)
Age 12	51	3–10	7.86 (1.30)	4139	0–10	8.36 (1.01)
Co-twin internalizing behavior						
Age 7	121	0–25	6.05 (4.99)	10,478	0–45	4.22 (4.31)
Age 10	98	0–43	6.40 (7.20)	8811	0–41	4.31 (4.71)
Age 12	87	0–26	5.44 (6.22)	6957	0–48	3.82 (4.47)
Co-twin externalizing behavior						
Age 7	123	0–34	7.53 (6.63)	10,616	0–46	5.95 (5.86)
Age 10	101	0–35	6.18 (6.17)	8876	0–53	5.28 (5.80)
Age 12	90	0–31	5.48 (6.17)	6999	0–50	4.30 (5.17)
Index child externalizing behavior						
Age 7	121	0–46	11.50 (9.52)	10,606	0–43	5.74 (5.78)
Age 10	100	0–42	10.59 (9.52)	8850	0–47	5.09 (5.64)
Age 12	88	0–27	9.32 (7.19)	6980	0–44	4.28 (5.13)
Parental well-being						
Mother						
Age 7	20	6–10	7.95 (0.94)	2763	1–10	7.91 (0.96)
Age 10	34	5–10	8.00 (1.04)	4260	0–10	7.93 (0.99)
Age 12	16	6–9	7.88 (0.81)	1658	2–10	7.91 (0.98)
Father						
Age 7	9	6–9	7.56 (0.88)	1479	3–10	8.01 (0.87)
Age 10	20	5–10	7.70 (1.26)	2504	0–10	7.94 (0.91)
Age 12	8	6–9	7.38 (1.19)	690	4–10	7.96 (0.86)
Parental monitoring						
Mother						
Age 7	17	10–12	11.53 (0.72)	1945	6–12	11.48 (0.86)
Age 10	29	6–12	11.38 (1.24)	3364	0–12	11.48 (0.93)
Age 12	39	7–12	11.26 (1.35)	2824	0–12	11.42 (1.15)
Father						
Age 7	10	9–12	11.00 (1.05)	1298	1–12	10.94 (1.41)
Age 10	7	6–12	11.14 (1.46)	798	3–12	10.99 (1.40)
Age 12	21	7–12	10.43 (1.94)	1825	0–12	10.30 (2.17)

^*^ The TD-group has not been used for age 5

### Preliminary Analyses

Based on the literature, we expected all demographic variables to influence the sibling outcome variables (Hayden et al., 2019; Wakimizu et al., 2020). To determine which of the demographic variables listed in Table 2 should be included in the analyses as covariates, we used simple regression to find which of the demographic variables were significantly related to the outcome variables. For the outcome variable externalizing behavior of the co-twin, all demographic variables (sex of the index child, sex of the co-twin, zygosity, year of birth, SES at age 10 and marital status at age 12) were significantly related for at least one age and hence included as covariates in the analysis with respect to this outcome variable. For internalizing behavior of the co-twin, only zygosity was not significantly related; for well-being of the co-twin, only the sex of the index child was not significantly related. The other variables are included as covariates in the corresponding analyses.

### Research Question 1: Change over Time

The results are summarized in Table 4. The high values for the ICC's reflect the need for, and justify the choice of, a multilevel model. An ICC of 0.77 for well-being shows that the variation in well-being of co-twins is made up for 77% of *between people variation* and for 23% of *within people variation* across time. For well-being and externalizing behavior of co-twins, m1 is the best model, where time (age) is modelled as fixed effect. This means that well-being and externalizing behavior of co-twins over time are best modelled by a line that describes the mean decrease in these outcome variables over time. There is no random component in m1, which shows that this is a constant effect that does not vary much per individual. The mean effect of age having the same sign for well-being and externalizing behavior may be counter intuitive. We address this issue in the discussion.

**Table 4:** Model of outcome variables over time

Outcome variable	# Twins	# Data points	ICC	Mean effect of time (*b*)	Best model
Well-being	87	127	0.77	−0.08^*^	m1
Externalizing behavior	169	314	0.71	−0.28^*^	m1
Internalizing behavior	168	306	0.65	−0.07	m2

^*^p < .05

For co-twin internalizing behavior there is no significant average effect of age and m2 turns out to be the best model, showing that internalizing behavior of co-twins over time is best described by a model that allows individual trajectories. Compared with externalizing behavior and well-being, internalizing behavior appears to change more per individual over time.

### Research Question 2: Group Difference and Mediation

For each outcome variable, the model was built up (from m0 to m3) in the same way as before, now using data where the ID-group (age 7–12) is merged with the TD-group (comparison group). The selected demographical variables were then added to the model as covariates and were removed again if not significantly related to the outcome variable. In the final step, we added the variable that distinguishes the ID-group from the TD-group. This predictor was only significant for the sibling outcome internalizing behavior, indicating that co-twins of children with ID have higher internalizing behavior than co-twins of typically developing children (first part of research question 2). The model fit was significantly better than that of the model without this variable (*X*^*2*^(1) = 20.8 *p* < 0.001). For co-twin well-being and co-twin externalizing behavior no significant group difference was found. The parameter information for the model of co-twin internalizing behavior is presented in Table 5.

**Table 5:** Parameter information (fixed effects) of multilevel model to predict internalizing behavior of co-twin with index child intellectual status (ID vs TD) as predictor (N = 14,257)

Variable	*b*	Standard Error *b*	95% Confidence Interval
Intercept	0.10	0.05	[0.002, 0.195]
Age	−0.05	0.01	[−0.072, −0.025]
Sex of co-twin	0.35	0.08	[0.188, 0.512]
Marital status	1.20	0.14	[0.922, 1.478]
SES	−0.20	0.04	[−0.280, −0.111]
Year of birth	−0.01	0.01	[−0.026, −0.002]
ID vs TD	1.82	0.40	[1.031, 2.600]

The random effects for this model are: *SD*(intercepts) = 3.04; *SD*(time) = 0.14; *r* = 0.75

**Fig. 2:**
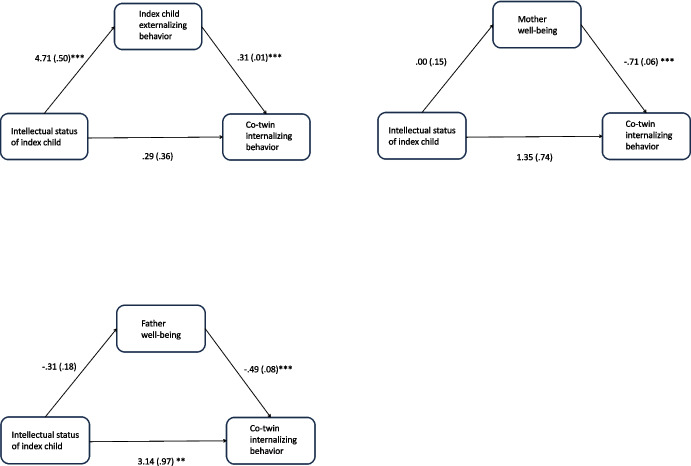
Mediation models with corresponding unstandardized beta regression coefficients and standard errors. **p < 0.01; ***p < 0.001

As co-twin internalizing behavior is the only outcome variable that is significantly predicted by the intellectual status of the index child, this is the only outcome variable that is investigated further in the mediation analysis. The investigated mediation models are shown in Fig. 2. A significant indirect effect *ab* is found for externalizing behavior of the index child (*ab* = 1.47, 95% CI [1.159, 1.775]), meaning that the effect of intellectual status of the index child on co-twin internalizing behavior is partly explained by the externalizing behavior of the index child. The percentage of the total effect that is mediated is 82%. Neither maternal well-being (*ab* = −0.001, 95% CI [−0.211, 0.203]) nor paternal well-being (*ab* = 0.152, 95% CI [−0.016, 0.346]) significantly mediated the relation between the intellectual status of the index child and co-twin internalizing behavior.

Because the behavioral problems of a child with ID and maternal well-being have been found to be related (Hastings et al., 2006; Neece & Baker, 2008), one could argue that, if a mediation model is tested with one of these variables as the mediator, the model should include the other variable as a covariate. When we rerun the previous analyses including this extra covariate, it appears that the main effect of the intellectual status of the index child is no longer significant when either one of these covariates is added. This is however no strict requirement for mediation and it turns out that there is still a mediating effect *ab* for externalizing behavior of the index child (*ab* = 1.33, 95% CI [0.6396, 2.035]). The percentage of the total effect that is mediated is 92%.

### Research Question 3: Moderation

Moderation analyses have been performed for all outcome variables (co-twin well-being, co-twin internalizing behavior and co-twin externalizing behavior) and predictors (child externalizing behavior, maternal well-being, paternal well-being) with maternal monitoring and paternal monitoring as possible moderators (research question 3). This analysis has been performed using the total dataset, taking the ID-group and TD-group together, because group differences were not significant when relevant covariates are included.

First, the models for each separate sibling outcome were tested including the predictors externalizing behavior of the index child, maternal and paternal well-being, and maternal and paternal monitoring. All were significant predictors of the three sibling outcomes (see Table 6). Covariates have been added to the model (and removed again when not significant) but are not shown in Table 6. Maternal well-being and maternal monitoring produce larger effects than paternal well-being and paternal monitoring. Externalizing behavior of the index child has a substantial effect on co-twin internalizing and externalizing behavior but only a small effect on co-twin well-being. For internalizing and externalizing behavior there is more variation in the model (larger random effects) than for well-being.

**Table 6:** Parameter information for three outcome variables in a multilevel multiple regression model with all relevant predictors

	Co-twin well-being (*N* = 7698)	Co-twin internalizing behavior (*N* = 14,257)	Co-twin externalizing behavior (*N* = 14,257)
*Fixed effects*			
Index child externalizing behavior	−0.02 ***	0.33 ***	0.62 ***
Well-being mother	0.39 ***	−0.50 ***	−0.29 *
Well-being father	0.06 **	−0.04	−0.25 *
Monitoring mother	0.08 ***	−0.23 *	−0.35 **
Monitoring father	0.03 **	−0.18 **	−0.21 **
*Random effects*	*SD*(intercept) = 0.50 *SD*(time) = 0.003 *r* = −0.01	*SD*(intercept) = 2.91 *SD*(time) = 0.005 *r* = 0.02	*SD*(intercept) = 3.50 *SD*(time) = 0.21 *r* = −0.86

Covariates are not shown

^*^p < .05 ^**^p < .01 ^***^p < .001

**Fig. 3:**
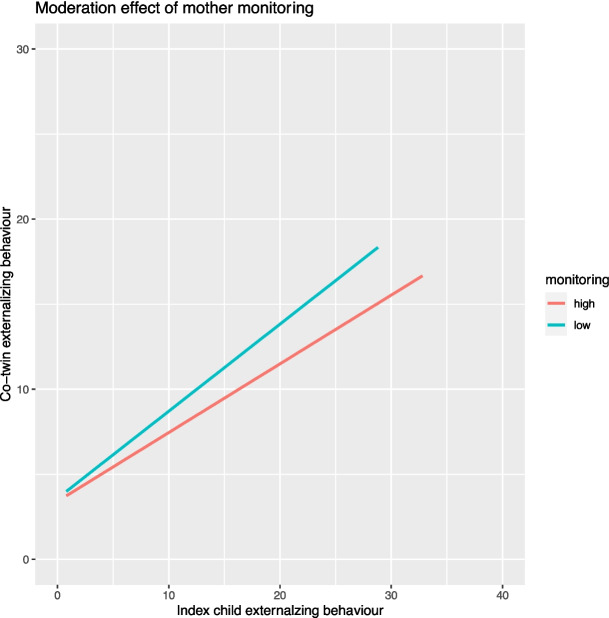
Moderation effect of mother monitoring on the relation between index child externalizing behavior and co-twin externalizing behavior

Next, separate moderation models were tested for interaction effects with maternal and paternal monitoring. Only maternal (but not paternal) monitoring was a moderator in the relation between index child externalizing behavior and co-twin externalizing behavior (*b* = −0.06, *p* < 0.01). This means that, for higher maternal monitoring, the relation between index child externalizing behavior and co-twin externalizing behavior is weaker (Fig. 3).

It is important to highlight that during construction of the models, the effects of several covariates changed from statistically significant to non-significant when externalizing behavior of the index child, parental well-being and parental monitoring were added to the model. In addition, the effect of age was not significant anymore when index child externalizing behavior was added. This means that the added predictor(s) correlate(s) with these variables and that some of the effect of the predictors was incorrectly attributed to the covariates. These non-significant covariates were removed from the model.

## Discussion

### Changes in Well-Being Over Time

In this longitudinal study of co-twins of children with or without ID, we focused on their well-being, internalizing and externalizing behavior. We modelled the change over time in these outcome variables and investigated mediation and moderation effects. Our first research question was: How does the well-being, internalizing and externalizing behavior of co-twins of children with ID change over time from age 5 to 12? We hypothesized that well-being would decrease over time and internalizing and externalizing behavior would increase. However, the average value of both well-being and externalizing behavior decreased slightly, whereas the average value of internalizing behavior did not change significantly. Although the average change of well-being and externalizing behavior over time were significant, there are two reasons why these results are not very meaningful. First, both well-being and externalizing behavior decreased over time, which is counter intuitive, as these concepts are usually seen as having opposite interpretations (i.e., the more externalizing behavior is observed, the lower the well-being). Second, in our later analyses, more covariates and predictors were added to the model, after which some covariates and also the factor of age did no longer significantly contribute to the model. This means that the predictors that were used in this study have a greater impact on the outcome variables than the effect of age. Future research could determine whether this also holds for non-twin siblings and for ages of siblings under 5 or above 12 years old.

### Mediating Effect of Externalizing Behavior

Our second research question was whether the intellectual status of a child (ID vs. TD) predicts the well-being and behavioral problems of their co-twin, and whether this relation is mediated by either the externalizing behavior of the child with ID or the well-being of the parents. We found that only the internalizing behavior of the co-twin is predicted by the intellectual status of a child. This indicates that internalizing behavior is a meaningful outcome measure for siblings of children with ID and contains elements on which these siblings differ from their peers. This finding aligns with other studies in which siblings were found to have elevated levels of internalizing behavior (Pinquart, 2023) and increased risk of depression (Martinez et al., 2022).

In the next step of the analysis, we found that child externalizing behavior mediates the relation between intellectual status of the index child and co-twin internalizing behavior. This means that the elevated levels of sibling internalizing behavior are not caused by the ID of the twin sibling, but by the consequential behavioral problems. This was found previously in the literature (Neece et al., 2010) and was hence confirmed with longitudinal data. When parental well-being was considered as a control variable, the main effect (i.e., the relation between index child intellectual status and co-twin internalizing behavior) was no longer significant, but the mediation effect was still present. We found no significant mediation for the models that include parental well-being as mediator. There are two aspects to highlight. First, although child externalizing behavior and parental well-being were previously found to have a bi-directional relationship (Hastings et al., 2006; Neece & Baker, 2008), only child externalizing behavior acts as an explanatory mediator. Second, if enough control variables are taken into account, there is no effect of child intellectual status on sibling outcomes, which tells us that all factors in which the ID-group differs from the TD-group have been taken into account. The fact that the demographic variables that are used in this study comprise only some of the factors that have been found to distinguish ID-families from TD-families shows that (at least some) factors are inter-related, as noticed previously (Marquis et al., 2019). An implication of this result is that there are different ways to mitigate the impact of externalizing behavior of a child on their sibling. A logical choice of support may be focused on reducing behavioral problems through intervention programs, but as factors appear to be inter-related, family support programs targeting more than one family member are encouraged and have been found to be effective (McKenzie Smith et al., 2018).

### Effect of Parental Monitoring

Our third and final research question was: Given any effects of externalizing behavior of the index child (with or without ID) and parental well-being on the sibling outcomes, is parental monitoring found to be a moderator? In this part of our analysis, we found that parental monitoring, index child externalizing behavior, and parental well-being are predictors of sibling well-being and behavior. In addition, we found that maternal monitoring is a moderator in the relation between index child externalizing behavior and co-twin externalizing behavior.

Parental monitoring was found to be related to higher well-being and lower internalizing and externalizing behavior of the co-twin. Although this specific measure of parental monitoring has not been used before in sibling studies, a few studies have included some measure on parenting. Giallo and Gavidia-Payne (2006) found that parenting and family factors are more important for predicting sibling adjustment than the siblings' own experience of stress and coping, and Platt et al. (2014) found that authoritative parenting was associated with cooperative behavior of the sibling. Both studies are in line with the present results.

We found a moderation effect for maternal, but not paternal, monitoring in the relation between index child externalizing behavior and co-twin externalizing behavior. Monitoring by the mother reduces the impact of externalizing behavior of a child on their co-twin's externalizing behavior. In combination with the found direct effects of maternal and paternal monitoring on all three sibling outcomes, this means that parental monitoring is important for and can influence sibling mental health and well-being. Therefore, this is advised to be stimulated by care organizations and included into programs for siblings and families. This result on parental monitoring provides support for one of the aspects of recommendations for sibling programs phrased as 'I need my parents' attention' (Marquis et al., 2022). The present study included only one aspect of parenting style, parental monitoring. It may be beneficial for future research to also include other aspects of positive parenting or parent–child relationship (Li, 2019; Sleddens et al., 2014) in order to gain a better understanding of the role of parenting for sibling mental health and well-being and possibilities for interventions in families regarding sibling outcomes.

### Additional Implications

Comparing the direct effects of the predictors on sibling outcomes, index child externalizing behavior has the greatest effect on co-twin externalizing behavior, whereas mother well-being has the greatest effect on co-twin internalizing behavior. This illustrates that different choices for sibling outcomes may tell different stories about the impact of predictors. Future research may compare these and other sibling outcome measures in order to reach consensus on the dimensions/indicators of sibling well-being. The fact that index child externalizing behavior has a large effect on co-twin externalizing behavior may be explained by the fact that children are known to sometimes imitate behavior (Barr & Hayne, 2010).

Index child externalizing behavior has a surprisingly small, though significant effect on co-twin well-being. The difference between this effect on co-twin well-being and internalizing behavior may be (partly) explained by the fact that well-being is a broader concept than internalizing behavior, that includes environmental factors as well (Patalay & Fitzsimons, 2016). In addition, the fact that co-twin well-being was measured with a single item only may also explain the small(er) effects of the predictors. The well-being of the mother has a reasonably large effect on co-twin well-being. However, because the well-being of the co-twin was also rated by the mother, these two measures of well-being may correlate which could explain this larger effect.

We found that parental well-being has an effect on co-twin externalizing behavior, and maternal (but not paternal) well-being has an effect on co-twin internalizing behavior. We can connect this with the literature, in which indicators of parental well-being have been found to impact sibling behavior and emotions. Koukouriki and Soulis (2020) found that parental anxiety positively relates to sibling anxiety, and Platt et al. (2014) found that caregiver burden is an indicator of externalizing behavior (but not when the model was controlled for by parenting style and sibling relationship). The fact that the effect on co-twin internalizing behavior was found for maternal well-being but not for paternal well-being may be explained by the finding that the siblings' relationship and communication with mothers are better than with fathers (Fredriksen et al., 2023) and by numbers showing that caregiving is done by mothers more than by fathers in this population (CBS, 2022). Future research is needed to confirm this explanation about the non-symmetrical effects of mothers and fathers.

### Limitations and Strengths

This study has a number of limitations, especially concerning the parental reports that were used. Mothers' ratings were used for internalizing/externalizing behavior and for well-being of the children. Mothers were considered to be good informants of their children's problem behavior (Loeber et al., 1990), however, 10–20% of the variance in parental ratings is accounted for by rater bias (Bartels et al., 2007b). In future studies, it may therefore be preferable to include both mothers' and fathers' ratings. Second, a rater contrast may be present, which means that a parent may rate the behavior of one child by taking the other child as a standard. As a result, parents may stress the similarities or differences between the children (Bartels et al., 2007a, b). Third, because mothers' reports were used to assess the child's well-being and mental health, as well as their own well-being, common method variance is a concern. Finally, discordance between sibling's and parent's reports has been found concerning sibling outcomes (Dinleyici et al., 2019; Guite et al., 2004) and suggests that self-reporting produces more reliable results. Self-reporting of well-being was not available for this age group in the YNTR, but it is available for the age group from 14 to 18 years and can be used in follow-up studies.

Another limitation is the fact that well-being (for co-twins, mothers and fathers) is measured by a single item. Although single-item measures are not necessarily less valid than multi-item measures (Allen et al., 2022), and the item that was used has a reasonable test–retest reliability, it is difficult to understand what was measured by this concept, especially as well-being is usually understood to be a broader concept than internalizing behavior (Patalay & Fitzsimons, 2016), the latter of which was measured using more items. However, studies have shown that the single item measure that was used is a good representation of well-being (Bartels & Boomsma, 2009; Baselmans et al., 2019).

Parental monitoring was measured by four items and had a mean Cronbach’s alpha of 0.61. The value of Cronbach’s alpha, measuring internal consistency, was higher for father ratings than for mother ratings and higher for measurements at older ages. These varying values question the construct validity of these items. Future research could evaluate and possibly adapt the items measuring parental monitoring. Furthermore, other possible measures of parental monitoring and parenting in general could be evaluated as moderators in research on siblings.

Another limitation addresses the notion of time in the mediation analysis. Gaynor (2017) suggests that outcome and process variables should be collected at different points in time in order to make a claim about causality in mediation analysis. Our data did not allow for this, since this method would leave us with too few data points to do the analysis. Therefore, no causality claims could be made from the mediation analysis in this study.

A final limitation of this study comes from the fact that we were not able to include all relevant variables according to the literature, such as for example the quality of the sibling relationship, because this variable is not measured in the YNTR dataset. The quality of the sibling relationship has been studied in families with a child with a disability and it was found to be associated with several sibling outcomes (Tomeny et al., 2017). Platt et al. (2014) mentioned that positive sibling relationships are more predictive of sibling outcomes than some parenting practices, and Wakimizu et al. (2020) even concluded that the sibling relationship is the most important of the investigated factors to influence the siblings' quality of life. Therefore, it is important for future research to also include the sibling relationship in longitudinal models of sibling outcomes.

The fact that our study on siblings was carried out from a twin perspective may have meaningful consequences. As twins were found to have a closer relationship than non-twin siblings (Fortuna et al., 2010), one may assume that the variation in the quality of the sibling relationship is lower among twins than among siblings in general. Therefore, the effect of sibling relationship on sibling well-begin may be smaller in a dataset of twin pairs than in a dataset of siblings in general. This means that the limitation of not including sibling relationship (mentioned above) may be less important in a study of twin pairs. Future research could further explore the role of sibling relationship in twin and non-twin sibling studies.

A strength of the data selection in our study may come from the fact that we used a very large database of twin pairs in which we identified children with ID ourselves. This minimized recruitment bias towards families with a special interest in the topic, as was mentioned earlier by Rum et al. (2022).

Given the specific dataset of Dutch twin pairs that we used, the results of this study are not directly generalizable to (twin-)sibling well-being and mental health in other cultures (Lauderdale-Littin & Blacher, 2016; Paul et al., 2021). Future research could replicate (parts of) our study in other countries and cultures.

### Conclusions and Implications

This study adds to the literature on the well-being and mental health of siblings of children with and without ID from a twin-family perspective. Several results from the literature were replicated in a longitudinal analysis. The three different sibling outcome measures showed different effects of the predictors, which implies that different studies using different outcome measures cannot easily be compared. We found that externalizing behavior of the index child, parental well-being and parental monitoring affects sibling well-being and mental health. The results imply that there are different ways to mitigate the impact of the externalizing behavior of a child on their sibling: by reducing externalizing behavior through interventions programs, by offering support to multiple family members, and by focusing on protective factors, such as parental monitoring. Future research should compare and organize sibling outcome measures in order to form consensus on indicators of sibling well-being and mental health. Furthermore, in addition to parental monitoring, other dimensions of positive parenting may be investigated to gain a better understanding of the role of parenting as a protective factor for sibling well-being and mental health.

## Data Availability

The data of the Netherlands Twin Register (NTR) may be accessed, upon approval of the data access committee, through the NTR (https://tweelingenregister.vu.nl/information_for_researchers/working-with-ntr-data).

## References

[CR1] Achenbach, T. M. (1991). *Child behavior checklist/4-18*. University of Vermont.

[CR2] Albores-Gallo, L., Lara-Muñoz, C., Esperón-Vargas, C., Zetina, J. A. C., Soriano, A. M. P., & Colin, G. V. (2007). Validity and reliability of the CBCL/6-18. Includes DSM scales. *Actas Españolas De Psiquiatría,**35*, 393–399. 18004676

[CR3] Allen, M. S., Iliescu, D., & Greiff, S. (2022). Single item measures in psychological science. *European Journal of Psychological Assessment,**38*(1), 1–5. 10.1027/1015-5759/a000699

[CR4] Arnold, C. K., & Heller, T. (2018). Caregiving experiences and outcomes: Wellness of adult siblings of people with intellectual disabilities. *Current Developmental Disorders Reports,**5*(3), 143–149. 10.1007/s40474-018-0143-4

[CR5] Barr, R., & Hayne, H. (2010). It’s not what you know, it’s who you know: Older siblings facilitate imitation during infancy. *International Journal of Early Years Education,**11*(1), 7–21. 10.1080/0966976032000066055

[CR6] Bartels, M., & Boomsma, D. I. (2009). Born to be happy? The etiology of subjective well-being. *Behavior Genetics,**39*(6), 605–615. 10.1007/s10519-009-9294-8 19728071 10.1007/s10519-009-9294-8 PMC2780680

[CR7] Bartels, M., Boomsma, D. I., Hudziak, J. J., van Beijsterveldt, T. C. E. M., & van den Oord, E. J. C. G. (2007a). Twins and the study of rater (dis)agreement. *Psychological Methods,**12*(4), 451–466. 10.1037/1082-989X.12.4.451 10.1037/1082-989X.12.4.451 18179355

[CR8] Bartels, M., van Beijsterveldt, C. E., Derks, E. M., Stroet, T. M., Polderman, T. J., Hudziak, J. J., & Boomsma, D. I. (2007b). Young Netherlands Twin Register (Y-NTR): A longitudinal multiple informant study of problem behavior. *Twin Research and Human Genetics,**10*(1), 3–11. 10.1375/twin.10.1.3 10.1375/twin.10.1.3 17539360

[CR9] Baselmans, B. M. L., van de Weijer, M. P., Abdellaoui, A., Vink, J. M., Hottenga, J. J., Willemsen, G., Nivard, M. G., de Geus, E. J. C., Boomsma, D. I., & Bartels, M. (2019). A genetic investigation of the well-being spectrum. *Behavior Genetics,**49*(3), 286–297. 10.1007/s10519-019-09951-0 30810878 10.1007/s10519-019-09951-0 PMC6497622

[CR10] Bauer, D. J., Preacher, K. J., & Gil, K. M. (2006). Conceptualizing and testing random indirect effects and moderated mediation in multilevel models: New procedures and recommendations. *Psychological Methods,**11*, 142–163. 10.1037/1082-989X.11.2.142 16784335 10.1037/1082-989X.11.2.142

[CR11] van Beijsterveldt, C. E., Groen-Blokhuis, M., Hottenga, J. J., Franic, S., Hudziak, J. J., Lamb, D., Huppertz, C., de Zeeuw, E., Nivard, M., Schutte, N., Swagerman, S., Glasner, T., van Fulpen, M., Brouwer, C., Stroet, T., Nowotny, D., Ehli, E. A., Davies, G. E., Scheet, P., . . . Boomsma, D. I. (2013). The Young Netherlands Twin Register (YNTR): Longitudinal twin and family studies in over 70,000 children. *Twin Research and Human Genetics*, *16*(1), 252–267. 10.1017/thg.2012.118 10.1017/thg.2012.118 23186620

[CR12] Biswas, S., Moghaddam, N., & Tickle, A. (2015). What are the factors that influence parental stress when caring for a child with an intellectual disability? A critical literature review. *International Journal of Developmental Disabilities,**61*(3), 127–146. 10.1179/2047387714Y.0000000043

[CR13] Blamires, J., Foster, M., Rasmussen, S., Zgambo, M., & Morelius, E. (2024). The experiences and perceptions of healthy siblings of children with a long-term condition: Umbrella review. *Journal of Pediatric Nursing,**77*, 191–203. 10.1016/j.pedn.2024.03.022 38574402 10.1016/j.pedn.2024.03.022

[CR14] Brown, S. M., Schlueter, L. J., Hurwich-Reiss, E., Dmitrieva, J., Miles, E., & Watamura, S. E. (2020). Parental buffering in the context of poverty: Positive parenting behaviors differentiate young children’s stress reactivity profiles. *Development and Psychopathology,**32*(5), 1778–1787. 10.1017/S0954579420001224 33427174 10.1017/S0954579420001224 PMC9118882

[CR15] Cantril, H. (1965). *Pattern of human concerns*. Rutgers University Press.

[CR16] CBS. (2022). Emancipatie monitor 2022: Werken en zorgen. https://longreads.cbs.nl/emancipatiemonitor-2022/werken-en-zorgen/

[CR17] Cebula, K., Gillooly, A., Coulthard, L. K. B., Riby, D. M., & Hastings, R. P. (2024). The experiences of children with williams syndrome and their nondisabled siblings of their relationship. *Family Relations*. 10.1111/fare.13102

[CR18] Chassin, L., Pillow, D. R., Curran, P. J., Molina, B. S. G., & Barrera, M., Jr. (1993). Relation of parental alcoholism to early adolescent substance use: A test of three mediating mechanisms. *Journal of Abnormal Psychology,**102*(1), 3–19. 10.1037/0021-843X.102.1.3 8436697 10.1037//0021-843x.102.1.3

[CR19] Dick, D. M., Viken, R., Purcell, S., Kaprio, J., Pulkkinen, L., & Rose, R. J. (2007). Parental monitoring moderates the importance of genetic and environmental influences on adolescent smoking. *Journal of Abnormal Psychology,**116*, 213–218. 10.1037/0021-843X.116.1.213 17324032 10.1037/0021-843X.116.1.213 PMC1807367

[CR20] Dinleyici, M., Çarman, K. B., Özdemir, C., Harmancı, K., Eren, M., Kirel, B., Şimşek, E., Yarar, C., Duyan Çamurdan, A., & Şahin Dağlı, F. (2019). Quality-of-life evaluation of healthy siblings of children with chronic illness. *Balkan Medical Journal,**37*(1), 34–42. 10.4274/balkanmedj.galenos.2019.2019.7.142 31647208 10.4274/balkanmedj.galenos.2019.2019.7.142 PMC6934013

[CR21] Dyke, P., Mulroy, S., & Leonard, H. (2009). Siblings of children with disabilities: Challenges and opportunities. *Acta Paediatrica,**98*(1), 23–24. 10.1111/j.1651-2227.2008.01168.x 19086940 10.1111/j.1651-2227.2008.01168.x

[CR22] Farrell, A. F., & Krahn, G. L. (2014). Family life goes on: Disability in contemporary families. *Family Relations,**63*(1), 1–6. 26185356 10.1111/fare.12053 PMC4501905

[CR23] Ferraioli, S. J., & Harris, S. L. (2009). The impact of autism on siblings. *Social Work in Mental Health,**8*(1), 41–53. 10.1080/15332980902932409

[CR24] Fortuna, K., Goldner, I., & Knafo, A. (2010). Twin relationships: A comparison across monozygotic twins, dizygotic twins, and nontwin siblings in early childhood. *Family Science,**1*(3–4), 205–211. 10.1080/19424620.2010.569367

[CR25] Fredriksen, T., Marie Vatne, T., Bjartveit Haukeland, Y., Tudor, M., & Fjermestad, K. W. (2023). Siblings of children with chronic disorders: Family and relational factors as predictors of mental health. *Journal of Child Health Care,**27*(1), 145–159. 10.1177/13674935211052157 34727780 10.1177/13674935211052157

[CR26] Gaynor, S. T. (2017). Temporal precedence in the identification of mediators of change: A brief comment on “mediators of change in the child/adolescent multimodal treatment study” (kendall et al., 2016). *Journal Consult Clinical Psychology,**85*(1), 77–79. 10.1037/ccp0000108 10.1037/ccp0000108 28045289

[CR27] Giallo, R., & Gavidia-Payne, S. (2006). Child, parent and family factors as predictors of adjustment for siblings of children with a disability. *Journal of Intellectual Disability Research,**50*(12), 937–948. 10.1111/j.1365-2788.2006.00928.x 17100954 10.1111/j.1365-2788.2006.00928.x

[CR28] Goodman, R. (1997). The strengths and difficulties questionnaire: A research note. *Journal of Child Psychology and Psychiatry,**38*(5), 581–586. 10.1111/j.1469-7610.1997.tb01545.x 9255702 10.1111/j.1469-7610.1997.tb01545.x

[CR29] Guite, J., Lobato, D., Kao, B., & Plante, W. (2004). Discordance between sibling and parent reports of the impact of chronic illness and disability on siblings. *Children’s Health Care,**33*(1), 77–92. 10.1207/s15326888chc3301_5

[CR30] Hastings, R. P., Daley, D., Burns, C., & Beck, A. (2006). Maternal distress and expressed emotion: Cross-sectional and longitudinal relationships with behavior problems of children with intellectual disabilities. *American Journal on Mental Retardation,**111*(1), 48–61. 10.1352/0895-8017(2006)111[48:Mdaeec]2.0.Co;2 16332156 10.1352/0895-8017(2006)111[48:MDAEEC]2.0.CO;2

[CR31] Hayden, N. K., Hastings, R. P., Totsika, V., & Langley, E. (2019). A population-based study of the behavioral and emotional adjustment of older siblings of children with and without intellectual disability. *Journal of Abnormal Child Psychology,**47*(8), 1409–1419. 10.1007/s10802-018-00510-5 30714074 10.1007/s10802-018-00510-5 PMC6616204

[CR32] Hayes, A. F., & Rockwood, N. J. (2020). Conditional process analysis: Concepts, computation, and advances in the modeling of the contingencies of mechanisms. *American Behavioral Scientist,**64*(1), 19–54. 10.1177/0002764219859633

[CR33] Honingh, A. K., Veerman, L. K. M., Bartels, M., & Sterkenburg, P. S. (2022). Wellbeing of co-twins of children with an intellectual disability: The role of parent factors and behavioural problems in a longitudinal analysis. In OSF preregistration. 10.17605/OSF.IO/XN8PV

[CR34] Hox, J. (2010). *Multilevel analysis: Techniques and applications* (2nd ed.). Quantitative methodology series. Routledge.

[CR35] Koukouriki, E., & Soulis, S. G. (2020). Self-reported health-related quality of life (HRQOL) and anxiety among greek school-age siblings of individuals with autism spectrum disorders (ASD) in relation to parental mental health and social support. *Journal of Autism and Developmental Disorders,**50*(8), 2913–2930. 10.1007/s10803-020-04395-6 32040799 10.1007/s10803-020-04395-6

[CR36] Kovshoff, H., Cebula, K., Tsai, H. J., & Hastings, R. P. (2017). Siblings of children with autism: The siblings embedded systems framework. *Current Developmental Disorders Reports,**4*(2), 37–45. 10.1007/s40474-017-0110-5 28680793 10.1007/s40474-017-0110-5 PMC5488140

[CR37] Lauderdale-Littin, S., & Blacher, J. (2016). Young adults with severe intellectual disability: Culture, parent, and sibling impact. *Journal of Intellectual & Developmental Disability,**42*(3), 230–239. 10.3109/13668250.2016.1230843

[CR38] Li, J. B. (2019). Parenting and self-control across early to late adolescence: A three-level meta-analysis. *Perspectives on Psychological Science,**14*(6), 967. 31491364 10.1177/1745691619863046

[CR39] Loeber, R., Green, S. M., & Lahey, B. B. (1990). Mental health professionals’ perception of the utility of children, mothers, and teachers as informants on childhood psychopathology. *Journal of Clinical Child Psychology,**19*(2), 136–143. 10.1207/s15374424jccp1902_5

[CR40] Marquis, S., Hayes, M. V., & McGrail, K. (2019). Factors that may affect the health of siblings of children who have an intellectual/developmental disability. *Journal of Policy and Practice in Intellectual Disabilities,**16*(4), 273–286. 10.1111/jppi.12309

[CR41] Marquis, S., O’Leary, R., Hayden, N. K., & Baumbusch, J. (2022). A realist review of programs for siblings of children who have an intellectual/developmental disability. *Family Relations,**72*(4), 2083–2102. 10.1111/fare.12789

[CR42] Martinez, B., Pechlivanoglou, P., Meng, D., Traubici, B., Mahood, Q., Korczak, D., Colasanto, M., Mahant, S., Orkin, J., & Cohen, E. (2022). Clinical health outcomes of siblings of children with chronic conditions: A systematic review and meta-analysis. *The Journal of Pediatrics,**250*(83–92), e88. 10.1016/j.jpeds.2022.07.002 10.1016/j.jpeds.2022.07.002 35810772

[CR43] McKenzie Smith, M., Pinto Pereira, S., Chan, L., Rose, C., & Shafran, R. (2018). Impact of well-being interventions for siblings of children and young people with a chronic physical or mental health condition: A systematic review and meta-analysis. *Clinical Child and Family Psychology Review,**21*(2), 246–265. 10.1007/s10567-018-0253-x 29450764 10.1007/s10567-018-0253-x PMC5899110

[CR44] Moyson, T., & Roeyers, H. (2012). “The overall quality of my life as a sibling is all right, but of course, it could always be better”. Quality of life of siblings of children with intellectual disability: The siblings’ perspectives. *Journal of Intellectual Disability Research,**56*(1), 87–101. 21366753 10.1111/j.1365-2788.2011.01393.x

[CR45] Neece, C., & Baker, B. (2008). Predicting maternal parenting stress in middle childhood: The roles of child intellectual status, behaviour problems and social skills. *Journal of Intellectual Disability Research,**52*(12), 1114–1128. 10.1111/j.1365-2788.2008.01071.x 18513339 10.1111/j.1365-2788.2008.01071.x PMC2787629

[CR46] Neece, C. L., Blacher, J., & Baker, B. L. (2010). Impact on siblings of children with intellectual disability: The role of child behavior problems. *American Journal on Intellectual and Developmental Disabilities,**115*(4), 291–306. 10.1352/1944-7558-115.4.291 20597724 10.1352/1944-7558-115.4.291

[CR47] Neece, C. L., Green, S. A., & Baker, B. L. (2012). Parenting stress and child behavior problems: A transactional relationship across time. *American Journal on Intellectual and Developmental Disabilities,**117*(1), 48–66. 10.1352/1944-7558-117.1.48 22264112 10.1352/1944-7558-117.1.48 PMC4861150

[CR48] Newland, L. A. (2020). Family well-being, parenting, and child well-being: Pathways to healthy adjustment. *Clinical Psychologist,**19*(1), 3–14. 10.1111/cp.12059

[CR49] Osborne, J. (2002). Notes on the use of data transformations. *Practical assessment, research, and evaluation*,* 8*(1), 6. 10.7275/4vng-5608

[CR50] Palmore, E., & Kivett, V. (1977). Change in life satisfaction: A longitudinal study of persons aged 46–70. *Journal of Gerontology,**32*(3), 311–316. 10.1093/geronj/32.3.311 850059 10.1093/geronj/32.3.311

[CR51] Patalay, P., & Fitzsimons, E. (2016). Correlates of mental illness and wellbeing in children: Are they the same? Results from the uk millennium cohort study. *Journal of the American Academy of Child and Adolescent Psychiatry,**55*(9), 771–783. 10.1016/j.jaac.2016.05.019 27566118 10.1016/j.jaac.2016.05.019

[CR52] Paul, A. M., Hussey, M. M., Woodman, A. C., Smith, A. L., & Shriver, T. P. (2021). Experiences of siblings of people with intellectual disabilities: Multiregional perspectives. *Family Relations,**71*(2), 671–685. 10.1111/fare.12608

[CR53] Pinheiro, J., Bates, D., & Team, R. C. (2023). nlme: Linear and nonlinear mixed effects models. In *R package version 3.1–162*. https://CRAN.R-project.org/package=nlme. Accessed June 2023.

[CR54] Pinquart, M. (2023). Behavior problems, self-esteem, and prosocial behavior in siblings of children with chronic physical health conditions: An updated meta-analysis. *Journal of Pediatric Psychology,**48*(1), 77–90. 10.1093/jpepsy/jsac066 35950954 10.1093/jpepsy/jsac066

[CR55] Platt, C., Roper, S. O., Mandleco, B., & Freeborn, D. (2014). Sibling cooperative and externalizing behaviors in families raising children with disabilities. *Nursing Research,**63*(4), 235–242. 10.1097/nnr.0000000000000046 24977720 10.1097/NNR.0000000000000046

[CR56] Ponterotto, J. G., & Ruckdeschel, D. E. (2007). An overview of coefficient alpha and a reliability matrix for estimating adequacy of internal consistency coefficients with psychological research measures. *Perceptual and Motor Skills,**105*(3), 997–1014. 18229554 10.2466/pms.105.3.997-1014

[CR57] Rum, Y., Genzer, S., Markovitch, N., Jenkins, J., Perry, A., & Knafo-Noam, A. (2022). Are there positive effects of having a sibling with special needs? Empathy and prosociality of twins of children with non-typical development. *Child Development,**93*(4), 1121–1128. 10.1111/cdev.13740 35194782 10.1111/cdev.13740

[CR58] Saxena, M., & Adamsons, K. (2013). Siblings of individuals with disabilities: Reframing the literature through a bioecological lens. *Journal of Family Theory & Review,**5*(4), 300–316. 10.1111/jftr.12021

[CR59] Selig, J. P., & Preacher, K. J. (2008). *Monte carlo method for assessing mediation: An interactive tool for creating confidence intervals for indirect effects [computer software]*. Available from http://quantpsy.org/. Accessed June 2023.

[CR60] Sleddens, E. F. C., O’Connor, T. M., Watson, K. B., Hughes, S. O., Power, T. G., Thijs, C., De Vries, N. K., & Kremers, S. P. J. (2014). Development of the comprehensive general parenting questionnaire for caregivers of 5–13 year olds. *International Journal of Behavioral Nutrition and Physical Activity,**11*(1), 15. 10.1186/1479-5868-11-15 24512450 10.1186/1479-5868-11-15 PMC3926334

[CR61] Tomeny, T. S., Ellis, B. M., Rankin, J. A., & Barry, T. D. (2017). Sibling relationship quality and psychosocial outcomes among adult siblings of individuals with autism spectrum disorder and individuals with intellectual disability without autism. *Research in Developmental Disabilities,**62*, 104–114. 10.1016/j.ridd.2017.01.008 28122290 10.1016/j.ridd.2017.01.008

[CR62] Totsika, V., Liew, A., Absoud, M., Adnams, C., & Emerson, E. (2022). Mental health problems in children with intellectual disability. *The Lancet Child & Adolescent Health,**6*(6), 432–444. 10.1016/S2352-4642(22)00067-0 35421380 10.1016/S2352-4642(22)00067-0

[CR63] Van Buuren, S. (2018). *Flexible imputation of missing data*. CRC Press.

[CR64] Wakimizu, R., Fujioka, H., Nishigaki, K., & Matsuzawa, A. (2020). Quality of life and associated factors in siblings of children with severe motor and intellectual disabilities: A cross-sectional study. *Nursing and Health Sciences,**22*(4), 977–987. 10.1111/nhs.12755 32662581 10.1111/nhs.12755

[CR65] Williams, C. A., Bailey, T., & Hastings, R. P. (2022). Modelling triadic relationships in families of children with intellectual disability. *Journal of Applied Research in Intellectual Disabilities,**35*(3), 843–855. 10.1111/jar.12988 35187793 10.1111/jar.12988 PMC9306971

[CR66] Wolff, B., Magiati, I., Roberts, R., Pellicano, E., & Glasson, E. J. (2022). Risk and resilience factors impacting the mental health and wellbeing of siblings of individuals with neurodevelopmental conditions: A mixed methods systematic review. *Clinical Psychology Review,**98*, 102217. 10.1016/j.cpr.2022.102217 36368218 10.1016/j.cpr.2022.102217

[CR67] Wolff, B., Franco, V. R., Magiati, I., Pestell, C. F., & Glasson, E. J. (2023). Neurocognitive and self-reported psychosocial and behavioral functioning in siblings of individuals with neurodevelopmental conditions: A study using remote self-administered testing. *Journal of Clinical and Experimental Neuropsychology,**45*(5), 513–536. 10.1080/13803395.2023.2259042 37779193 10.1080/13803395.2023.2259042

[CR68] Yaldız, A. H., Solak, N., & Ikizer, G. (2021). Negative emotions in siblings of individuals with developmental disabilities: The roles of early maladaptive schemas and system justification. *Research in Developmental Disabilities,**117*, 104046. 10.1016/j.ridd.2021.104046 34388576 10.1016/j.ridd.2021.104046

[CR69] Yaremych, H. E., Preacher, K. J., & Hedeker, D. (2021). Centering categorical predictors in multilevel models: Best practices and interpretation. *Psychological Methods*. 10.1037/met0000434 34914468 10.1037/met0000434

[CR70] Zarra-Nezhad, M., Moazami-Goodarzi, A., Aunola, K., Nurmi, J.-E., Kiuru, N., & Lerkkanen, M.-K. (2019). Supportive parenting buffers the effects of low peer acceptance on children’s internalizing problem behaviors. *Child & Youth Care Forum,**48*(6), 865–887. 10.1007/s10566-019-09510-y

